# A second demographic transition in Indonesia?

**DOI:** 10.1007/s42379-022-00115-y

**Published:** 2022-10-21

**Authors:** Ariane Utomo, Aris Ananta, Diahhadi Setyonaluri, Calvin Aryaputra

**Affiliations:** 1 School of Geography, Earth and Atmospheric Sciences, The University of Melbourne, Melbourne, Australia; 2 Faculty of Economics and Business, Universitas Indonesia, Depok City, Indonesia; 3 The Centre for Advanced Research, Universiti Brunei Darussalam, Gadong, Brunei Darussalam; 4 Population and Labour Studies, Faculty of Economics and Business, Universitas Indonesia, Depok City, Indonesia; 5 Faculty of Economics and Business, Universitas Indonesia, Depok City, Indonesia

**Keywords:** Indonesia, Fertility, Marriage, Family, Second demographic transition, Islam, Diversity

## Abstract

As a predominantly Muslim and ethnically diverse new democracy in Asia, Indonesia is a timely case to study how the contending forces of development and social change are reflected in changing norms and practices around family formation. This paper examines the extent to which the second demographic transition (SDT) theory can provide a primary framework to understand contemporary patterns of fertility, marriage and family change in Indonesia. Against the backdrop of socio-political change following *Reformasi in* 1998, we found emerging demographic features typically associated with societies in later stages of fertility transition. These include fertility below replacement in some regions; increasing age at first marriage, non-marriage, and divorce rates; and growing diversity in household/family forms. As the vast regions of Indonesia is economically, culturally, and demographically heterogeneous, these key features of SDT are not likely to emerge and unfold in a uniform manner. Further, these demographic shifts are taking place amidst multiple tensions and contradictions in the nature and direction of ideational change pertaining to marriage and the family. We argue that the prevailing *ideational change* driving the shifts in marriage, fertility, and the family within Indonesia is neither unilinear nor singular in nature. Emerging ideational change embodying individualism, secularism, and post-materialism—originally proposed in SDT theory to be the primary drivers of fertility decline in post-industrial Western Europe—can overlap with popular values promoting de-secularization and the strengthening of familial institutions. As a demographic framework, the SDT theory is an important and useful starting point. But it needs to be reevaluated by considering the complex socio-political and increasingly precarious economic terrains behind fertility transition, as well as marriage and family change in post-*Reformasi* Indonesia.

## Introduction

Based on the empirical contexts of industrialization in the Western world, the demographic transition theory, later called “first demographic transition” in this paper, was once the dominant framework to explain the association between socioeconomic transformations and demographic change across different parts of the world. In brief, the (first) demographic transition theory outlines the process and drivers of shifts from high to low death and birth rates, and the ensuing changes in the pace of population growth through the course of such shifts. It was hypothesized that by the end of the demographic transition, fertility and mortality would reach replacement level usually measured with Total Fertility Rate (TFR) around 2.1 (Kirk, 1996; Lesthaeghe, 2014).^1^ It is a condition of a hypothetical equilibrium between number of births and deaths after constant fertility and mortality rates for about 40 years. It should be mentioned that the hypothetical equilibrium does not necessarily mean good or bad. Furthermore, drawing from the first demographic transition theory, the expectation in the early 1970s was that by the end of the first transition, households all over the world would converge to a nuclear and conjugal type, consisting of a married couple and their children. However, this equilibrium was not observed, at least in Europe (Lesthaeghe, 2014).

By the latter half of the twentieth century, it became apparent that the first demographic transition theory could no longer offer a sufficient framework to understand the continuing decline in fertility to below replacement levels in some parts of Europe. In 1986, the term “Second Demographic Transition” was coined by two European demographers–Dirk van de Kaa and Ron Lesthaeghe–as a novel framework to understand key features of demographic change in the Western world (see Lesthaeghe, 2010; Kaa, 1987).

The Second Demographic Transition theory underlines the importance of ideational change behind shifts in fertility to below replacement level, and the accompanying shifts in marriage and family trends. In particular, it has been argued that the SDT theory could provide a comprehensive framework to account for a series of interrelated features in contemporary demographic phenomena in the West, including delayed marriage and parenthood, sub-replacement fertility, the rise of alternative forms of partnerships (including cohabitation), and parenthood outside of marriage (Lesthaeghe, 2020).

Lesthaeghe (2010) observed that emerging features of the SDT coincided with an ethics revolution marked by changes in society’s values from social orientation to self-actualization, with more emphasis on individual rights over community/family needs, as well as rising secularism and demand for democracy. Zaidi and Morgan (2017, p. 573) summarized that the SDT theory is centered on the proposition that “a powerful, inevitable, and irreversible shift in attitudes and norms in the direction of greater individual freedom and self-actualization” will drive a “unilinear change towards very low fertility and a diversity of union and family types.” As one of the key theoretical concepts on contemporary fertility decline in European societies (Graham, 2021; Han & Brinton, 2022), the SDT framework has been adopted by a large number of empirical studies in other parts of the Western world and, more recently, in parts of Asia.

This paper aims to provide insights into contemporary patterns of fertility transition and the associated shifts in marriage and family patterns in Indonesia, and the extent to which the SDT framework can provide a primary analytical lens to understand such trends. Indonesia offers a unique and important case study to theorize how the contending forces of development and social change are reflected in changing norms and practices around family formation in a predominantly Muslim, ethnically diverse new democracy in Asia.

Relative to the demographic experiences in other Southeast Asian countries such as the Philippines and other countries in West and East Asia, Indonesia provides an important empirical and theoretical case to examine the applicability of the SDT beyond the context of the West. On the surface, Indonesia’s experience with fertility decline is relatively similar to the experience in other Muslim-majority countries in the Middle East and North Africa region, where countries like Egypt, Iran and Turkey have reached below replacement fertility levels (see Pourreza et al., 2021). As the nation with the world’s largest Muslim population, the socio-political, cultural, and religious contexts of demographic change in Indonesia are particularly of interest to this special issue; and even more so in light of Lesthaeghe’s recent suggestion that the absence of major cultural shifts and the persistently high share of women with low education in countries with a Muslim or Hindu tradition are factors that inhibit the increase in cohabitation—one marked feature of the SDT (Lesthaeghe, 2020).

In this paper, we begin by briefly outlining two key challenges that would ascertain the extent to which the concept of SDT can be applied to the current demographic and socio-political landscapes of Indonesia. Here, we argue that the first challenge is to do with diversity in the trends around fertility, marriage, and family change across the diverse regions and population sub-groups in Indonesia. That is, as Indonesia is socially, culturally, economically, and demographically heterogeneous, the decline of total fertility rate to below replacement level—a key feature of SDT—is unlikely to unfold in a uniform manner across the archipelago.

Second, we point to the growing diversity in public discourses, norms, attitudes, and practices around marriage and family formation, particularly following the onset of the *Reformasi* period in 1998. *Reformasi* began following the end of over three decades of the autocratic rule of President Suharto and his New Order Regime (1966–1998). Along with significant shifts to decentralized governance, *Reformasi* unleashed a wide array of “alternative” ideas around marriage and family formation, including those emerging with the growing popularity of what Ariel Heryanto (2011) calls Islamic chic/pop Islam among urban educated young adults (also see Smith-Hefner, 2005, 2018, 2019). These are likely to continue to challenge (a) the decades-long dominance of the State-sponsored discourses pushed by the National Family Planning Program, and (b) the hypothesis that secularization and individualization would spearhead a single and linear shift in marriage and family formation ideals and outcomes.

Against this backdrop of demographic diversity and socio-political change following *Reformasi*, the following empirical sections of our paper juxtapose key features of the second demographic transition theory with contemporary trends and variations in fertility, marriage, and the family in Indonesia. Sections 3 and 4 draw on prevailing trends in fertility, marriage, and household typologies respectively. Drawing on a range of datasets, these sections are guided by the following questions:

i. Has Indonesia as a whole—or certain parts of Indonesia/sub-groups of the population—reached below replacement level of fertility?
ii. Have there been significant transformations in patterns of family formation and the institution of marriage?

In Sect. 5, we further canvas the growing diversity of norms and practices on gender, work, marriage, and family formation since the onset of *Reformasi*. We discuss the extent to which the intersecting forces of democratization, religion, and globalization are driving these trends across the vast and diverse Indonesian archipelago. Here, we further highlight how social media has amplified the visibility of family diversity in recent years. We speculate how this has challenged and changed the dominant narrative of the ideal family which had been heavily promoted under the Family Planning program during New Order Indonesia.

In our concluding remarks, we discuss how the case of Indonesia showcases that ideational change in marriage and family formation in the post-Reformasi context is characterized by tensions and contradictions. The ensuing ideational change is neither unilinear (as argued by Zaidi & Morgan, 2017) nor singular in nature and, as such, is likely to result in variegated features across multiple dimensions of the second demographic transition. We argue that the discourse and practices surrounding marriage and family formation among Indonesian young adults are quite distinct from those found in East Asia. In addition, the case of Indonesia diverges from the often-touted assumptions of the nature of ideational change and demographic transition in the Muslim world. We show that despite a general trend of fertility decline, even at below replacement level of fertility in some areas, and a growing trend in delayed and non-marriage, marriage remains a largely common ideal among young people in contemporary Indonesia.

## Background: diversity in post-*Reformasi* Indonesia

**Fig. 1:**
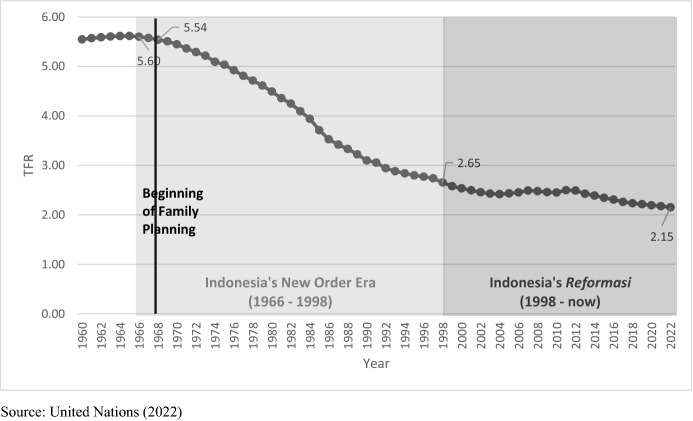
Fertility decline in Indonesia total fertility rate 1966–2020. Source: United Nations (2022)

Between the late 1960s and the late 1990s, Indonesia experienced a sharp decline in fertility Since then, the fertility decline had plateaued and with TFR hovering above replacement, at about 2.6 children per woman during 2002–2012 (Fig. 1). Contraceptive use had been rising—though slowly—during 2002–2012 (National Population and Family Planning Board, Statistics Indonesia, Ministry of Health, and the DHS Program, 2018). Fertility has started to decline again in 2014.

The official projection/target of the government of Indonesia (Badan Pusat Statistik, 2018), the 2022 United Nations World Population Prospects (United Nations, 2022), and our own calculation (described in Sect. 3 of this paper) indicate that Indonesia may have been at replacement level of fertility, with TFR at about 2.1 in 2022 (the time this paper was written). Nevertheless, it is not easy to answer whether the second demographic transition concept/theoretical framework can be applied to understand the broader forces of societal change that underpin such a transition. In outlining the country-specific backdrop to examine the empirical fit of the SDT framework for Indonesia, we argue for the need to center two underlying notions of *diversity* when assessing contemporary patterns in fertility, marriage, and family change in the country.

The first of these is demographic diversity. While often touted as home to the largest Muslim population in the world, Indonesia is a large heterogenous archipelagic country, with many regions, religions, ethnic groups, languages, cultures, and kinship systems. The 2010 Indonesian Population Census provides over 1300 codes for ethnic categories, with certain ethnic categories/sub-categories being coterminous with religion. These codes were then refined and reclassified to produce more than 630 ethnic groups (Ananta et al., 2015; Arifin et al., 2015). Further, the vast Indonesian archipelago is currently sub-divided into 34 provinces and a further 514 district-level governments with varying stages of economic development and demographic transition. Pre-COVID-19 pandemic data have suggested a marked variation in the period TFR at the sub-national level, with some regions already showing below replacement fertility.

As shown in Tables 1 and 2 in Sect. 3, we argue that Indonesia as a whole may have been below replacement level in 2020, with some provinces/ethnic groups still above the replacement level, and some others much below replacement level. As such, examining trends in marriage and family formation requires an explicit acknowledgment of the diversity of the speed, direction, and drivers of such trends across the many regions and population sub-groups in Indonesia.

**Table 1:** TFR and wanted TFR by provinces: Indonesia, 2014–2017

Provinces	Wanted TFR		TFR	
	2009–2012	2014–2017	2009–2012	2014–2017
Sumatera				
Aceh	2.1	2.5	2.8	2.7
North Sumatera	2.3	2.4	3.0	2.9
West Sumatera	2.2	2.0	2.8	2.5
Riau	2.1	2.4	2.9	2.9
Jambi	1.7	2.0	2.3	2.3
South Sumatera	2.3	2.2	2.8	2.6
Bengkulu	1.8	2.0	2.2	2.3
Lampung	2.3	2.0	2.7	2.3
Bangka Belitung	2.1	1.8	2.6	2.3
Riau Islands	1.8	1.9	2.6	2.3
Java				
Jakarta	1.8	1.8	2.3	2.2
West Java	1.9	2.1	2.5	2.4
Central Java	2.1	2.1	2.5	2.3
Yogyakarta	1.8	1.8	2.1	2.2
East Java	2.0	1.8	2.3	2.1
Banten	1.9	2.1	2.5	2.3
Bali and Nusa Tenggara				
Bali	1.9	1.6	2.3	2.1
West Nusa Tenggara	2.2	2.2	2.8	2.5
East Nusa Tenggara	2.5	2.9	3.3	3.4
Kalimantan				
West Kalimantan	2.3	2.3	3.1	2.7
Central Kalimantan	2.2	2.1	2.8	2.5
South Kalimantan	2.0	2.1	2.5	2.4
East Kalimantan	2.0	2.1	2.8	2.7
North Kalimantan		2.4		2.8
Sulawesi				
North Sulawesi	1.8	1.8	2.6	2.2
Central Sulawesi	2.2	2.2	3.2	2.7
South Sulawesi	1.8	2.1	2.6	2.4
Southeast Sulawesi	2.3	2.3	3.0	2.8
Gorontalo	1.9	2.1	2.6	2.5
West Sulawesi	2.5	2.4	3.6	2.7
Maluku and Papua				
Maluku	2.4	2.6	3.2	3.3
North Maluku	2.2	2.4	3.1	2.9
West Papua	1.9	2.6	3.7	3.2
Papua	2.4	2.8	3.5	3.3
Indonesia	2.0	2.1	2.6	2.4

Source: compiled from ICF (2022). We note the controversy surrounding the TFR estimates from the last three rounds of the Indonesian Demographic and Health Surveys due to the undercounting of unmarried women and the ensuing potential overestimation of TFRs

**Table 2:** Stage of population aging by ethnic group: Indonesia 2010

State of population aging in 2010		% of population to total national population	Ranking of ethnic group in terms of number of population	TFR in 2000–2005
Transitional	Javanese	40.06	1	1.96
8.0–12.0%	Madurese	3.03	5	2.08
	Balinese	1.66	11	
	Chinese	1.2	15	
	**Total**	**45.95**		
Youthful	Sundanese	15.51	2	2.55
6.0–8.0%	Minangkabau	2.73	7	
	Buginese	2.71	8	
	Acehnese	1.44	12	
	Sasak	1.34	14	
	**Total**	**23.73**		
Very young	Malay	3.7	3	2.86
< 6.0%	Batak	3.58	4	3.18
	Betawi	2.88	6	
	Bantenese	1.96	9	
	Banjarese	1.74	10	
	Dayak	1.36	13	
	**Total**	**15.22**		
	Grand total	**84.9**		

Source: compiled from Ananta et al. (2005) and Ananta et al. (2015)

The bold numbers refers to the total percentage in each group of the category of transitional, youthful, and very young

The second is to do with diversity in the nature and direction of ideational change related to norms, attitudes, and practices around marriage and family formation following *Reformasi*. Much of the scholarship on fertility transitions in Indonesia has focused on the role of the family planning program that began in the 1970s (Hull, 2016; Jones, 2005; McDonald, 2014; Reese, 1975). During the reign of Suharto’s New Order government in 1966–1998, the National Family Planning program—*Keluarga Berencana*—actively promoted the ideals that (1) two children are enough; and (2) the ideal ages at marriage for men and women were 25 and 20 respectively. Such social marketing was deemed successful in changing the ideation of family size from “many children bring fortunes” to the norms of small family size.^2^

Heaton and Cammack (2011) noted how values actively promoted in the Family Planning Program were steeped in development idealism (Thornton, 2001; Thornton et al., 2012, 2015). Developmental idealism is defined as “…a cultural model—a set of beliefs and values—that identifies the appropriate goals of development and the ends for achieving these goals” (Thornton et al., 2015, p. 277). In this context, parallel to the centering of ideational change in the SDT framework, developmental idealism under the New Order saw fertility decline and the associated change in family norms as an integral part of development. Here, along with norms promoted through the Family Planning program, women’s advancement in education and their increasing participation in paid employment go hand-in-hand as main drivers of increasing age at first marriage and childbirth during the New Order era (Hull, 2003, 2016; Jones, 2005, 2007).

**Fig. 2:**
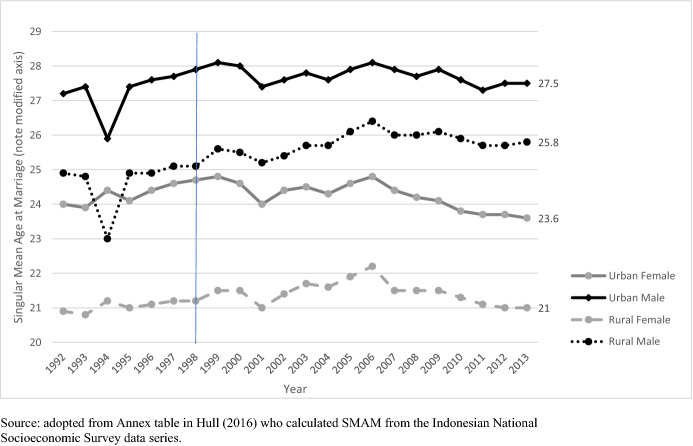
Reversal in singular mean age at marriage: 1992–2013

In 1998—at the end of Suharto’s 32 years of autocratic rule—democratic euphoria introduced an air of optimism for more egalitarian gender relations in Indonesia. Simultaneously, this period of *Reformasi* also provided a platform for the re-emergence of *repressive* customs and religious laws and heavily patriarchal stereotypes, and ethno-religious sentiments, in both national and local politics (Afrianty, 2019; Utomo, 2016). In the decades following *Reformasi*, there has been growing speculation concerning the return of early marriage in Indonesia (see Grijns et al., 2019). This is somewhat reflected in available data. For example, Hull (2016) points to the reversal of trends of singular mean age at marriage (SMAM): the female SMAM rose between 1990 and 2005 and fell between 2005 and 2010 comparable to the 2000 level (Fig. 2). This reversal has been attributed to a religion-motivated ideational shift that encourages and romanticizes early marriage.

In a Muslim majority country, childbirth—let alone sex or cohabitation—outside of marriage remains collectively shunned. Social media mediated campaigns by organizations such as Indonesia without dating (*Indonesia Tanpa Pacaran*) have actively promoted early(er) marriage among educated young adults to avoid sinful pre-marital relationships (Sari et al., 2020). This story of contested values and conflicting trends surrounding gender roles, marriage, fertility, and the family is an emerging feature of the post-New Order era in Indonesia (see Platt et al., 2018). An example of such narrative of contested values is the controversy around the 1974 Marriage Law.

In the 1974 Indonesian Marriage Law, with parental consent, the minimum ages for women and men to get formally married were sixteen and nineteen respectively. Furthermore, the stipulation in the 1974 Marriage Law that marriages would fall under the auspices of religious authorities also served to discourage and inhibit interreligious marriages (Aini et al., 2019). The 1974 Marriage Law had been effective in discouraging child marriage in parts of Indonesia during the reign of the New Order. However, recent legal challenges in the Constitutional Court have sought to revise the minimum legal age of marriage upwards and to revoke the law that inhibits interreligious marriage. The push to revise the legal marriage age had been predominantly framed as a way to end child marriage (Grijns et al., 2019). In 2015, the Indonesian Constitutional Court rejected a petition to review the minimum legal age of marriage for women. Amidst public debate, at the end of 2019, the Constitutional Court ruled that the minimum age of marriage for girls as set out in the 1974 Marriage Act was unconstitutional. Following the ruling in 2019, the minimum age for men and women to get married without parental consent has been revised to nineteen.^3^ Without parental consent, individuals need to be at least 21 to register their marriages. While the campaign to revise the legal age at marriage has been successful, more recent legal petition for interreligious marriage remains unsuccessful.^4^

Controversies surrounding the 1974 Marriage Law stand as examples of ongoing contestations on how the state, parents, kinship networks, and religious authorities continue direct who can get married and when. In the past, the New Order Regime pushed for a set of hegemonic norms around marriage, family, and fertility under and beyond its Family Planning Program. In contrast, *Reformasi* has provided a platform for multiple non-state actors and authorities to shape competing public discourses on these issues.

In this manner, the demographic question of who, when, and how individuals get married becomes a unique entry point to capture the fraught processes of ideational change in the country. Within the public discourses around family formation, emerging ideas around individualization and secularization—which are among the noted pillars of ideational change proposed in the SDT framework—have continued to materialize in the midst of what others had called a *conservative turn* (Bourchier, 2019; Van Bruinessen, 2013) and heightened Islamic piety in post-*Reformasi* Indonesia (Smith-Hefner, 2019).

In sum, the diverse landscapes of demographic and ideational change briefly outlined in this section provide a timely context for us to rethink the extent to which the framework of SDT might be applied to Indonesia in particular, and to the Muslim world more broadly.^5^ We will return to this point after the next two sections, where we outline key characteristics in Indonesia’s fertility transition and accompanying shifts in broad marriage/family formation patterns.

## Below replacement fertility?

### Fertility by provinces

As discussed in Badan Pusat Statistik (2018), the official “population projection” has two scenarios for the year 2045. The first scenario is based on the government’s official policy that Net Reproduction Rate (NRR) should never be below 1 (should never be below replacement level). NRR is targeted to be equal to 1 in 2020 and to remain at 1 until 2045. In provinces with NRR below 1, the policies are to stop the declining trend and even reverse the trend to raise the fertility rate. However, there has been no official explanation why NRR = 1 should be the target or for an “ideal” population trajectory, if there exists any “ideal” population trajectory. The first scenario has been used as the official population “projection”, meaning population targets/policies. Therefore, under the official policy stance, Indonesia is not *allowed* to go below replacement level or, perhaps, prohibiting the possibility of this central feature of the *SDT*.

The second scenario is following a projected trend. It also assumes NRR = 1 or Total Fertility Rate (TFR) = 2.1 in 2020, but the TFR then slowly declines toward 1.97, just slightly below replacement level, in 2045. In this projection, fertility is allowed to be below replacement level, but will never go deeper. There has not been any official explanation of why fertility is not *allowed* to decline further. This second scenario is similar to the United Nation World Population Projection 2022 for Indonesia.^6^ They only allowed TFR to eventually go down to 1.91, not much below replacement level of fertility.

These two scenarios are in stark contrast to one of the predictions of SDT that all countries will eventually reach sustained below replacement level. Our analysis provides a different picture from the government official “projection” and the “trend” scenario by the Government and the United Nations mentioned earlier. The government uses data from censuses/intercensal surveys which usually provide lower estimates of fertility than those based on Demographic and Health Surveys (DHS) data. Our analysis is based on the more conservative estimates based on DHS. Based on the 2017 DHS (ICF, 2022), TFR in Indonesia as a whole declined from 3.0 during 1988–1991 to 2.6 during 1999–2003. It then remained constant for about a decade, at 2.6 until 2009–2012. It then declined again, reaching 2.4 in 2014–2017. That means Indonesia was still above replacement level of fertility in 2014–2017. It was still in the First Demographic Transition (FDT), but in the last stage of FDT, defined in this paper as TFR below 2.5 but above 2.1 (see Table 1).

Moreover, as also shown in Table 1, the wanted TFR was already 2.1, at replacement level. With TFR at 2.4 during 2014–2017 and wanted TFR at 2.1 during the same period, we therefore project that TFR in Indonesia as a whole may have been at replacement level, or even just below replacement level in 2022 (at the time this paper was prepared).^7^ This is similar to the official policy and estimated trend from the government of Indonesia. However, unlike the official policy, we argue that the declining trend may continue and, unlike the UN’s estimates, the TFR may eventually go down much lower than 1.9.

As we outlined earlier, Indonesia is a large and diverse country. It has 34 provinces with a relatively large variations in their development levels and TFRs.^8^ At present, two of Indonesia’s 34 provinces were already at replacement level (with TFR = 2.1) during 2014–2017. They were the provinces of East Java and Bali. East Java is one of the home provinces of the Javanese, the largest ethnic group in Indonesia. Bali is the home province of the Balinese, the 11th largest ethnic group in Indonesia. Some districts within the two provinces may have had below replacement fertility levels for some time (BKKBN, 2022). These two provinces’ fertility rates have been below replacement level for longer than Indonesia as a whole.

TFRs in 13 provinces were already as low as 2.2, 2.3, or 2.4, very near to replacement level, the last stage of the FDT, during 2014–2017. They were Jambi, Bengkulu, Lampung, Bangka Belitung, and Riau Island (in Sumatera Island); Jakarta, West Java, Central Java, Yogyakarta, and Banten (in Java Island); South Kalimantan (in Kalimantan Island); and South Sulawesi and North Sulawesi (in Sulawesi Island). Under prevailing trends, these 13 provinces are projected to enter below replacement fertility rate. Altogether, there were already 15 out of 34 provinces that had been or were very close to below replacement during 2014–2017.

Furthermore, wanted TFR in eight provinces (West Java, Central Java, Banten; Central Kalimantan, South Kalimantan, and East Kalimantan; South Sulawesi and Gorontalo) were already at replacement level. There were 12 provinces which already had wanted below replacement level: West Sumatera, Jambi, Bengkulu, Lampung, Bangka Belitung, Riau Islands; Jakarta, Yogyakarta, East Java, and Banten; Bali; North Sulawesi. Even Bali’s wanted TFR was already at 1.6, well below replacement level.

This may indicate that in more than half of the provinces. The wanted fertility rate may have been below replacement levels in 2014–2017. In other words, 20 out of 34 provinces may have been below replacement level in 2022. It is possible that all provinces in Java Island are at or below replacement level in 2022. The population on Java Island accounts for about 55.5% of the total population in Indonesia. In contrast, there were four provinces with TFR still above 3.0 during 2014–2017. They were East Nusa Tenggara, Maluku, West Papua, and Papua (all in Eastern Indonesia). Their wanted TFR remained high, above 2.5 (but already below 3.0). Therefore, these four provinces are still far from entering the below replacement level.

In short, the majority of provinces may have been at or below replacement level in 2020. Only few are still above or far above (near 3.0) the replacement level. This pattern supports our argument that declining fertility below replacement level may continue.

### Fertility by ethnicity

Indonesia’s demographic diversity can also be seen from its ethnic groups. Indonesia had more than 630 ethnic groups in 2010 (Ananta et al., 2015). TFR may also vary by ethnic group. However, data on fertility by ethnicity is very limited. The only available estimate is Ananta et al. (Ananta et al., 2005), though limited to the five largest ethnic groups (Javanese, Sundanese, Malay, Batak, and Madurese), using data from the 2005 intercensal population census. However, there is information on the age structure of the 15 largest ethnic groups in Indonesia in 2010 (Ananta et al., 2015). The researchers classified whether the age structure of the population was in the transitional stage of population aging (with a percentage of 60+ between 8 and 12%), in the youthful stage (with a percentage of 60+ between 6 and 8%), or the very young stage (with a percentage of 60+  < 6%).

We have data on TFR below replacement level by the five largest ethnic groups in 2000–2005 from Ananta et al. (2005). Two of them, Javanese and Madurese, were already at or below replacement level. At the same time, based on Ananta et al. (2015), they were in the transitional stage of population aging in 2010. Therefore, we use the transitional stage of population aging as a proxy for at or below replacement level. Table 2 shows the stage of population aging in each of the 15 largest ethnic groups in Indonesia in 2010, followed by the percentage of each ethnic group, as well as the TFRs of the five largest ethnic groups in Indonesia (Javanese, Sundanese, Malay, Batak, and Madurese) during 2000–2005.

First, Table 2 shows that 45.95% of the total population was already in the transitional stage in 2010. There were four ethnic groups in this stage: Javanese, Madurese, Balinese, and Chinese. They might have been below replacement level in 2010. The Javanese, the largest and most dominant ethnic group in Indonesia, was already in the transitional stage in 2010. Their TFR was 1.96 in 2000–2005. Madurese was also in the transitional stage and its TFR was 2.08 in 2000–2005. The Madurese is close to Javanese, with their home in the Island of Madura, part of Java Island and the Province of East Java. The other two ethnic groups who were also in the transitional stage of population aging were the Balinese and Chinese. These two groups were not included in Ananta et al. (2005) and we therefore do not have their TFRs. The percentage of older people among the Chinese was even closer to the upper boundary of the transitional stage (12%). It is likely therefore that the Chinese may have been in the old state (percentage of 60+  > 12.0%) by 2022—they may have been far below replacement level. In other words, almost half of the population in Indonesia in 2010 may have been below replacement level.

Table 2 also reveals that almost a quarter of the population was already in the youthful stage of population aging. They may be in the last stage of FDT. They may have been or are near to below replacement level of fertility in 2022. In contrast, 15.22% of the population was still in the very young stage of population aging in 2010. They were less likely to have been below replacement level in 2022. They are the Malay, Batak, Betawi, Bantenese, Banjarese, and Dayak. Note that we only discuss the 15 largest ethnic groups in Indonesia, though these groups account for 84.9% of the total population in Indonesia.

The data presented in this section suggests that the TFR of major ethnic groups and certain provinces—whose population makes up more than half of the country—may already have reached below replacement fertility levels in 2022. While noting the existence of Indonesia’s demographic diversity, we speculate that Indonesia as a whole may be at or below the replacement level of fertility in 2022. Some provinces and ethnic groups may be well below the replacement level, and some others remain above replacement level. Moreover, fertility may continue declining below replacement level. Once again, we underline that it is never easy to discuss “Indonesia”: which *Indonesia* are we referring to when discussing fertility transition?

## Marriage and family change

As a Muslim majority population, marriage and having children have remained near universal ideals and practices across much of Indonesia. In this section, we examine whether there have been significant transformations in patterns of family formation and in the institution of marriage using multiple demographic data sources. Given the absence of data on variables such as cohabitation rates, we focus on other key variables indicative of the proposed underlying family formation features under the second demographic transition. These are: age at first marriage, age at first sexual intercourse, marital status, and living arrangements.

### Age at first marriage

The United Nations (2019) shows that the mean age at first marriage for Indonesia has been relatively low compared to other ASEAN countries. The figure for Indonesia increased from 19.3 to 22.4 during 1970–2017, while the Philippines, for example, was 22.8 in 1970 and 23.4 in 2017. In comparison to more developed Asian economies, the number was significantly lower; Japan’s and South Korea’s were 29.2 and 31.5, respectively. It is also lower than the figures for Europe (27.2 in 2010) and North America (24.6 in 2010) (Hertrich, 2017).

**Fig. 3:**
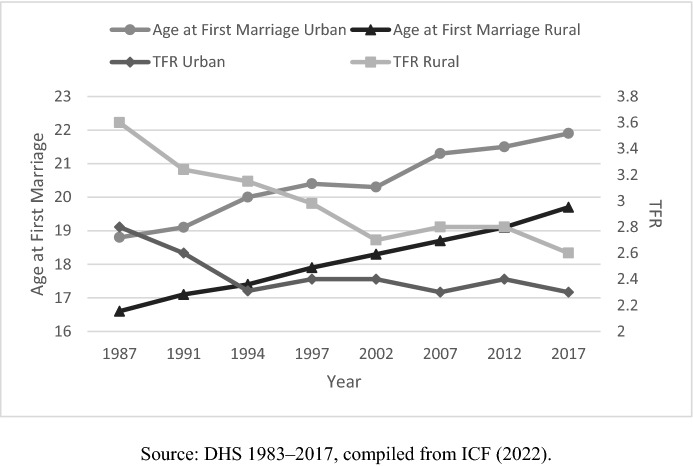
Median age at first marriage and TFR, 1983–2017

Despite there being advancement in women’s education and an increase in female labor force participation since the end of the 1980s (Najib et al., 2021; Rumble et al., 2018), the trend of delayed marriage in Indonesia has not been as striking as in other neighboring countries over the past two decades. The median age at first marriage among women aged 25–49 years increased by only 3 years from 17.2 in 1987 to 20.8 years in 2017 (ICF, 2022). Contrary to the earlier expectation that improved education is associated with delayed marriage, the data shows a persistent trend of marrying young across education levels: The median age at first marriage among women with secondary and higher education was stagnant at 22.3 years between 2007 and 2017, while women with lower education experienced a slight increase in age at first marriage, from 18 to 18.4 years during the same period (ICF, 2022). Figure 3 outlines the trends in median age at first marriage and TFR for urban and rural Indonesia using data derived from the DHS series.

Regional variation in age at first marriage in Indonesia is correlated with variation in the prevalence of child marriage. On the whole, despite the rising age at first marriage, UNICEF (2021) suggests that child marriage—that is, marriage prior to the age of 18—in Indonesia is still relatively high. It is 16.0% among women aged 20–24 who were first married before age 18. It is much higher than the 7.0% in East Asia and the Pacific and 10.0% in the world. The rate of child marriage is higher among women residing in rural areas, and at the lower end of the socioeconomic and education spectrum. However, the trend shows that child marriage’s prevalence has declined with age groups, indicating that child marriage may have reduced across generations despite the rate still being high compared to other countries.

Central Kalimantan and South Kalimantan had the lowest age at first marriage in 2017, standing at 19 and 19.5, respectively. This is because both provinces had the highest prevalence of child marriage in 2017. According to the National Socioeconomic Survey (SUSENAS), the percentage of women aged 20–24 whose age at first marriage was under 18 in Central Kalimantan was 20.94%, while in South Kalimantan it was 23.12%. On the other hand, the figures for Riau Islands, Jakarta, and Yogyakarta were the highest at 23.1 in 2017. Similarly, it was due to the low prevalence of child marriage: the figure for Riau Islands was 4%, Jakarta was 3.18%, and Yogyakarta was 2.21%. The huge variation in median age at first marriage between provinces is largely attributed to significant differences in cultures, religions, and social norms (Rumble et al., 2018).

Aceh’s median age at first marriage shows a significant increase from 17.1 in 1991 to 21.9 in 2017. Following *Reformasi*, Aceh has been noted as one of the most conservative provinces in Indonesia and since 2001, its local government has enforced Sharia Law, which forbids unmarried couples spending time together. Under this law, those who are found to have had intimate relationships outside of marriage will be publicly caned. Due to the restrictive environment, people are reluctant to date, reducing the likelihood of being married. However, Aceh has a high prevalence of *nikah siri*—which is an unofficial or unregistered marriage officiated by religious leaders—so the figures for age at first marriage may be biased.

### Age at first sexual intercourse

According to DHS (ICF, 2022), the median age at first sexual intercourse for women aged 25–49 has been increasing, from 17.8 in 1991 to 20.9 in 2017. Identical to the median age at first marriage, the lowest numbers were attained by Central Kalimantan (19.4) and South Kalimantan (19.5) in 2017, while the figures for Riau Islands (23.4), Jakarta (23.4), and Yogyakarta (23.3) were the highest. This indicates a similarity between age at first marriage and age at first sexual intercourse, with a very small percentage of pre-marital sexual intercourse.

The 2015 Global School-based Health Survey (GSHS) revealed that 6.9% of male and 3.8% of female students in Indonesia had ever had sex. Determinants of sexual intercourse among students include gender, school performance, substance use (alcohol, drugs, and tobacco), school truancy, suicidal ideation, parental supervision, and peer support (Rizkianti et al., 2020).

In 2017, the median age at first marriage for Indonesian men (24.6) was higher than the median age at first sexual intercourse (24.2), indicating that some men are exposed to pre-marital sexual intercourse. However, we should be cautious in interpreting these data, as people may be reluctant to say when they had their first sexual intercourse if it happened outside marriage, especially in Indonesia where pre-marital sexual intercourse is seen as taboo. O’Donnell et al. (2020), using the 2010 Greater Jakarta Transition to Adulthood Survey (GJTAS), showed that after considering evidence of underreporting, the percentage of pre-marital sex by age 25 years for women increased from 3.5% (without accounting for potential underreporting) to 18%.

### Marital status

In contrast to Asian countries that face SDT with a declining married population over time, the marriage trend in Indonesia indicates that Indonesians continue to lean towards marriage as a universal norm. In 2017, the majority of women aged 15–49 were currently married (71.5%), with the highest percentage (92.0%) at ages 35–39 (ICF, 2022). The percentage of married individuals in Indonesia is far higher compared to Japan, where 69.8% of women aged 35–39 were married in 2015 (Statistics Bureau of Japan, 2015), and even compared to the Philippines, where 65.4% of women aged 35–39 were married in 2017 (ICF, 2022).

Although it is practiced in some regions in Eastern Indonesia (Buttenheim & Nobles, 2009), cohabitation or living together is not an acceptable norm and is subject to social sanctions (Himawan et al., 2018). National Population Family Planning Board et al. (2018) reported that the percentage of people aged 15–49 living together was small, at around 0.4%, with the highest (0.8%) in ages 20–24, followed by 0.7% in ages 25–29. The percentage living together is smaller the older is the age group, except for the youngest group, 15–19, which is 0.3%. In contrast, almost all men aged 15–54 in relationships were legally married (99.6%), with the rest cohabitating. A relatively high percentage of cohabitation (1.5%) was observed among men aged 20–24.

Meanwhile, the available data shows that there is an anomaly in the divorce pattern in Indonesia. Although it is acceptable, the social norms discourage divorce, particularly among Muslims, and stigmatize female divorcées (Widyastari et al., 2020). DHS shows that the trend of divorce has been relatively low at around 3% between 1987 and 2017. After it reached its lowest rate of 2.5% in 1997, the percentage of divorced women aged 15–49 years experienced an upturn to 2.8% in 2017 (ICF, 2022). The surge in the divorce rate is also observed in Islamic court data after *Reformasi.* Muslim divorce cases in 2014 increased by 2.6 times in comparison with 2001, and 70% of the cases were initiated by women (Qibthiyyah & Utomo, 2016). Although early marriage and duration of marriage continue to be two strong factors attributed to divorce, greater autonomy of women in terms of spouse selection and decreased dependency on spouses were proposed as explanations for the higher likelihood of divorce in Indonesia (Heaton & Cammack, 2011; Widyastari et al., 2020).

Similar to divorce, despite being single or never married being associated with a derogatory social status in Indonesia, data shows that rates of remaining single have been increasing over the past decade (Himawan et al., 2018).^9^ Setyonaluri et al. (2020) estimated that the share of never-married population in the age group 40–65 years rose from 4.3 to 5.3% between 2007 and 2017. The rise of singlehood, combined with delayed marriage and widowhood, leads to changing patterns of living arrangements in Indonesia, particularly a surge in one-person households.

### Living arrangements

The one-person household has been a rising phenomenon in Asia. Apart from the widowed population, the rise in one-person household in the region has been primarily attributed to the rising percentage of young and middle-aged urban adults who live alone because of delayed or declining marriage, divorce, and wider geographical mobility The share of one-person household in Japan, South Korea and Taiwan rose from 19.8%, 4.8% and 11.8% in 1980 to 32.4%, 23.9%, and 22% respectively (Yeung & Cheung, 2015). The growing share in one-person household has also been observed in other parts of Asia, albeit at much lower levels than in East Asia (Yeung & Cheung, 2015).

Our analysis from the Indonesian National Socioeconomic survey series suggests that Indonesia is not an exception. Table 3 shows there have been rising trends in one-person households, couples without children, and one parent households with children. The percentage of single-person households rose from 6.03% in 1993 to 8.03% in 2020; couples without children, from 7.43% to 9.40%; and one parent with children, from 5.52 to 7.39%. On the other hand, Table 3 indicates the continued dominance, though declining, of the extended family (couple with children and other relatives) and the nuclear family (couple with children). The percentage of two-parent families with children declined from 55.14% in 1993 to 50.48% in 2020. The second-largest percentage is seen in the extended family, with a rising trend from 9.57% in 1993 to 11.58% in 2020.

**Table 3:** Share of household types, 1993–2020. Source: National Socioeconomic Survey (SUSENAS) 1993, 2000, 2010, and 2020

Household types	1993	2000	2010	2020
One person household	6.03	5.76	6.32	8.03
Couple without children	7.43	8.74	8.58	9.4
Couple with children	55.14	56.41	51.4	50.48
One parent with children	5.52	6.12	6.39	7.39
Couple without children and relatives	1.47	1.31	1.77	1.3
Couple with children and relatives	9.57	9.48	12.02	11.58
One parent with children and relatives	2.79	2.94	4.51	4.74
Relatives living together (no family nucleus)	1.01	1.03	1.21	1.17
Couple without children and relatives and non-relatives	0.26	0.22	0.22	0.15
Couple with children and relatives and non-relatives	1.68	1.26	1.33	1
One parent with children and relatives and non-relatives	0.27	0.28	0.39	0.34
Relatives and non-relatives living together	0.47	0.35	0.3	0.32
Head and others	1.56	1.52	1.23	1.34
Couple without children and others	0.9	0.69	0.61	0.46
Couple with children and others	5.4	3.47	3.25	1.96
One parent with children and others	0.51	0.42	0.44	0.36
Total	100	100	100	100

The above table shows an increasing trend of non-*traditional* forms of family in Indonesia. People in these categories are often viewed negatively in Indonesia. Single women may be stigmatized and labeled as “*perawan tua*” or spinsters. They are perceived as someone who has poor self-esteem, is incompetent, too selective, or self-oriented (Himawan et al., 2018; Setyonaluri et al., 2020; Situmorang, 2007). Even if they have a solid career or education, unmarried people are frequently regarded as “incomplete” (Situmorang, 2007). Muslims in Indonesia [around 87.18% of the Indonesian population (Badan Pusat Statistik, 2011)] also believe that marriage is a religious obligation. People often consider childfree families as abnormal or assume they have poor relationships, while single parents, especially divorcées, are negatively stereotyped as problematic people. The increasing proportion of these household types may imply that there is a gradual shift from the traditional culture and norm (ethics revolution) in Indonesia.

In addition, increasing singlehood might also be caused by improving education and female labor force participation. According to Statistics Indonesia (BPS), the mean years of schooling for men increased from 7.91 to 8.92, while for women they rose from 6.89 to 8.17 during 2010–2021 (Badan Pusat Statistik, 2022b). The trend also shows that women have outnumbered men in attaining tertiary education. The percentage of women aged 15 and older who completed tertiary education increased from 6.13 to 10.06%, while men increased from 6.67 to 9.28% between 2009 and 2021 (Badan Pusat Statistik, 2022a, 2022c). Women’s participation in formal employment has also been increased from 43 to 50% between 2010 and 2021 although overall female labour force participation rate had been stagnant at around 50% during the same period (Cameron et al., 2019).

Studies show that better education and participation in paid employment for women have changed the timing and nature of marriages in Indonesia. As women become more educated and financially independent, women have a higher autonomy of when and who to marry including the decision to stay single permanently, and increase requirements for partners (assortative mating), which increases the likelihood of being single (Dykstra & Poortman, 2009; Ntoimo & Isiugo-Abanihe, 2014). On average, educated women expect to marry men with the same or higher level of education (Malhotra 1997), with equal values of shared domestic work and childcare (Quah 1998). As a consequence, women face an increasing ‘difficulties’ of finding spouse who fulfill their requirements and extend the period of singlehood (Himawan et al., 2018; Hull, 2002).

## SDT and contested narratives of ideational change under *Reformasi*

How do the trends in fertility decline and marriage and family change in Indonesia stand relative to the demographic features proposed by the SDT? The analysis in Sects. 3 and 4 can be summed up as follows. First, there are already indications of sub-replacement fertility in certain regions in Indonesia that cover significant segments of the population, with national fertility at the replacement level in 2020. We also argue that fertility will keep declining, even though rates have already been below replacement level. Second, while there is a longer-term trend indicative of delayed entry into first marriage, recent data is suggestive of either a slowing down in the rise of—or even slight reversal of—age at first marriage. Third, while marriage remains the most dominant demographic status in adulthood, there is a growing share of non-marriage, as well as rising rates of divorce. Fourth, data on living arrangements are suggestive of the declining dominance of nuclear and extended family, and correspondingly, the emergence of diverse family and household typologies. Lastly, while there is no reliable national data on their prevalence, pre-marital sexual intercourse and cohabitation may be rare.

Given these somewhat inconclusive trends, can we say that Indonesia is approaching its second demographic transition? The original SDT framework underlines the importance of a certain kind of ideational change behind sub-replacement fertility. More precisely, the SDT is characterized by societal shifts towards individualism and post-materialism. So, is there evidence of such ideational change behind fertility transition in post-*Reformasi* Indonesia?

First, we propose that patterns in Indonesia’s fertility transition can be partly explained by the shifts in ideational forces caused by the abrupt end of the authoritarian New Order and the ensuing contestation of ideas following *Reformasi*. As we outlined in the Background section, the push for family planning and the associated hegemonic family values promoted by the New Order had facilitated a sharp decline in fertility. But, notwithstanding the regional variation, fertility decline in the country as a whole has slowed down since the early 2000s. Furthermore, similar to trends in neighboring countries like Vietnam and Thailand, and even the Muslim majority Malaysia, the national TFR was at or slightly below replacement level in 2020, with a prospect of further decline. Such trends are consistent with the proposition that the influence of multiple ideational forces may have helped to maintain *marriage and having children* as near universal ideals following *Reformasi*. Indeed, despite the bourgeoning literature on Indonesia’s conservative turn and the associated rise of political Islam, we are not seeing any significant spikes in fertility, nor are we seeing a significant reversal in age at first marriage.

Accounting for such contestations of ideational forces is particularly important when assessing other elements in reproductive rights and outcomes in post-Reformasi Indonesia. For example, although it is underreported, it is important to acknowledge the role of induced abortion on declining trend of TFR in Indonesia. Guttmacher Institute (2020) reported that an estimated 1.7 million abortions took place in Java in 2018, corresponding to a rate of 43 abortions per 1000 women aged 15–49, compared to 34 for Southeast Asia. A most recent estimate using Confidante Method in 2020 found that abortion rate was 42 per 1000 women in Java, which is a stark contrast compared to estimate of abortion rate from the direct report at 3.4 per 1000 women (Stillman et al., 2020). The lack of exploration of induced abortion and fertility is affected by the negative stigma around abortion. Induced abortion is legally restricted in Indonesia. Abortion is allowed under medical emergencies and in cases of rape although it is limited up to six weeks of gestation. Social norms continue to stigmatize induced abortion particularly among unmarried women. And regulations surrounding abortion have been subjected to intense debates during the revision process of the national penal codes.

Second, to understand how democratic reforms are shaping ideational change pertaining to marriage and the family, we argue that one must resist the temptation toward binary categorization. This is often the case in many developmental paradigms (Zaidi & Morgan, 2017). *Reformasi* unleashed a plethora of ideational forces, actors, and authorities. Some might be tempted to label these—and the associated norms and values that they promote—along a spectrum: conservative on the one end, and progressive on the other. But in practice, these diverse actors represent complex and shifting alliances among activists, religious authorities, and civil society organizations, as well as opportunistic politicians and oligarchic powers. Furthermore, these *seemingly contradictory forces* might often coalesce, producing *seemingly contradictory trends* that stand against the stereotypical portrayal of the Islamic world (particularly of women).

As an example, while growing calls for *halal love*—broadly meaning discouragement of premarital relationships including courtship—might play a role in the slight reversal in the long-run trend of delayed entry into marriage, we are not seeing any concerning setbacks in gender parity in education. Indonesia does not fit the problematic assumption that large segments of women in Muslim countries tend to have relatively low education levels. In fact, data from the latest population census 2010 indicated that for every 100 women aged 25–29, there were only 76 men in the corresponding educational category (Qibthiyyah & Utomo, 2016). This is in stark contrast to the experience of the older cohorts among those aged 50–54 in 2010, the sex ratio within the tertiary-educated population was 176 men to 100 women. More recent data suggest that although the mean years of schooling for women (8.7) was still lower than for men (9.23) in 2021, the percentage of women aged 15 and over completing tertiary education (10.06%) was higher than the corresponding figure for men (9.28%) (Badan Pusat Statistik, 2022a, 2022c).

As another example, the bourgeoning industry of Islamic fashion suggests that many urban young middle-class Muslim women in Indonesia can both be devout Muslims while exhibiting postmaterialist inclination of self-expression and creativity (Beta, 2014). Further, despite the increasing popularity of the *hijab* among the younger and educated segment of the population, this does not stop their engagement in and enthusiasm for political and environmental causes (Beta, 2019; Nilan, 2021). In the same way, the popular set of ideals of what makes a good young Muslim family is imbued with small family norms, and postmaterialist values stressing the importance of travel and leisure and a sense of community (*the Ummah*) (Rakhmani, 2016, 2019).

Third, we propose that social media plays a major role in facilitating multiple ideational forces relevant to fertility transition after *Reformasi*. As is the case in other parts of the world, social media platforms such as Instagram, WhatsApp, Facebook, TikTok, and Twitter are immensely popular among young adults in Indonesia (DataReportal, 2022). On the one hand, social media have amplified the visibility of discourses promoting Islamic or pious family values (see Smith-Hefner, 2019; Nisa, 2021). On the other hand, social media also have facilitated emerging discourses on singlehood, childfree, and non-heteronormative lifestyles. In other words, the proliferation of ideas, values, norms, and practices concerning gender roles, marriage, the family, and ultimately fertility in Indonesia has been driven by the intersecting forces of democratization, globalization, and the revolution in ICT technology. A case in point here is the discourse on polygamy (see Nurmila, 2009). Although the reporting and promotion of polygamous marriage based on a particular interpretation of Islamic practices through numerous media/social media channels had been amplified following Reformasi, it is not—by far—the dominant or even "popular" ideals in marriage/family options among young adults.

In sum, the contending forces directing ideational change on marriage and the family have presented a challenge to the singular narrative of fertility and family ideals of the New Order. Ideational change under *Reformasi* is no longer singular nor unidirectional in nature. As articulated by Heryanto (2011, p. 76), the case of post-*Reformasi* Indonesia suggests that “modernity does not imply or require secularization”. This is an important contribution to future research aiming to theorize the variegated nature and direction of ideational change pertaining to fertility transition.

## Concluding remarks: future pathways

In light of the contentious terrains of ideational change under *Reformasi*, what might be the future outlook for the fertility transition in Indonesia? We speculate that much of this will be underpinned by two factors: what is happening in the realm of gender, work, and the family; and the impact of the increasing economic precarity for young people brought by the changing nature of work.

First, despite achieving remarkable expansion in education for women, the pervasiveness of patriarchal norms continues to inhibit the progress of gender equality in Indonesia. The female labor force participation rate in Indonesia was stable at around 50% from 2010 to 2020 (Cameron et al., 2019), and is lower than in neighboring countries like Vietnam and Thailand (World Bank, 2021). Marriage and childbearing have a strong negative association with female labor force participation in Indonesia. Although participation in paid work is deemed positive, when they work, women are assumed as secondary earners and adjust their work around their domestic responsibilities (Setyonaluri et al., 2022; Utomo, 2012). Previous studies have suggested that the internalization of gender norms is influenced by religious beliefs that view women’s ideal role as a mother and carer for the family (Setyonaluri et al., 2022). The mental and physical load for working women is augmented by urbanization and small family norms. These have been accompanied by a decline in reliance on familial networks for childcare.

For educated women in urban areas, the privatized/familial support model for childcare has been made possible by an excess pool of women from rural areas with lower education levels being available to work as live-in domestic helpers (Nasution, 2020). But with the continued expansion of education across the country, this is unlikely to be sustainable. Given the relative scarcity of childcare provision outside the home, it remains to be seen whether the persistence of patriarchal norms and the associated role incompatibility with women’s economic participation is likely to further push fertility below replacement level in the coming years. This is particularly important given the increasing pressure on dual-earning families under current labor market conditions for young people.

The current context of gender equality brings the question about the future fertility in Indonesia. Borrowing McDonald (2000)’s terminologies in his Gender Equity Theories of Fertility Transition, Indonesia has progressed in gender equality in individual-oriented social institutions, as indicated by increase of women’s education and female’s participation in formal sector employment. However, inequality seems to exist in the family-oriented social institutions as married women continue to exit the labour market following marriage and childbearing as the result of persistent strong male-breadwinner norms in Indonesia. The direction of fertility transitions will depend on how women navigate the conflict between their roles at home and public ‘achievement’ of advancement in education and career prospect.

Second, while economic and housing precarity have been theorized as key factors behind fertility transition in East Asia (Fukuda, 2020; Raymo et al., 2015), we have yet to see concrete data of a similar phenomenon unfolding in Indonesia. Indonesia has a large segment of workers in the informal sector, and like other lower middle-income countries, its social security system remains largely absent. In recent decades, the labor market prospects of a large segment of educated young people in the Global South—including Indonesia—have been plagued by a series of constraints. These include premature deindustrialization (Rodrik, 2016), global trends towards casualization, and the rise of non-standard work (Mills & Blossfeld, 2013). A recent study on fertility preferences in Indonesia noted that the economic rationale has continued to drive young adults’ desire for a two-child family, and the negative gradient between fertility ideals and education seems to have been upended (Utomo et al., 2021). With the added impacts of the prolonged COVID-19 pandemic, it might be the case that ideational change pertaining to marriage and fertility in Indonesia will be even more constrained by heightened labor force uncertainties.

In sum, the second demographic transition theory was first proposed in the 1980s to explain the transition to below replacement fertility in a specific historical context in Western Europe. Most recently, in light of varying structural and ideational forces that drive the global fertility transition, Lesthaeghe (2020) reiterated that a single-factor explanation can no longer suffice. By making qualitative assessments of the contending ideational forces behind the prevailing patterns of fertility transition in Indonesia, our paper supports Lesthaeghe’s observation. Following democratic reforms in the late 1990s, we show that even within a country, fertility-relevant ideational change is multifaceted and multidirectional in nature. By examining fertility, marriage, and the family in Indonesia through the starting lens of the SDT, this paper provides a foundation for comparison to other nations in Asia and the Muslim world, enhancing our understanding of the complex and multifaceted nature of socio-demographic change in these diverse and rapidly transforming regions.

## Footnotes

1 We refer to period TFR in this paper. While TFR is often translated to the average number of children per woman in the media, we remind readers of its definition: in a given year, period TFR is “(t)he number of children a woman would have if she experienced the age-specific fertility rates for the period in question throughout her reproductive life.” (http://papp.iussp.org/sessions/papp101_s04/PAPP101_s04_080_030.html). Period TFR is an artificial measure based on a synthetic cohort of women, and caution should be applied when interpreting the estimates in our paper.

2 There had been debates whether the decentralisation of the family planning program following Reformasi in 1998 led to TFR being “stuck” above replacement level. Hull (2016, p.137) argued that data issues with DHS estimates should be considered, and that policy makers should not jump to conclusions of the National Family Planning Board’s failure to address a “population explosion”.

3 Detailed revisions to the 1974 Marriage Law can be accessed here: https://jdihn.go.id/files/4/2019uu016.pdf.

4 In February 2022, the Constitutional Court rejected a request for a judicial review for the Marriage Law to allow for interfaith marriage See https://www.thejakartapost.com/culture/2022/02/09/catholic-man-asks-for-marriage-law-review-after-failing-to-marry-muslim-partner.html.

5 It should be noted here that some provinces in Indonesia have non-Muslim majority population, such as Christianity or Hinduism.

6 See https://population.un.org/dataportal/data/indicators/19/locations/360/start/1950/end/2022/table/pivotbylocation.

7 The wanted TFR was actually already at 2.0 in 2009–2012. The rise of wanted TFRs along with declining TFRs in some provinces may mean the existence of high cost in achieving the wanted TFR. Further studies should examine this phenomenon.

8 There is a corresponding variation in contraceptive use across regions. In 2017, the current use of contraceptive methods by all women ranges from 28% in Papua up to 56% in Central Kalimantan has the highest prevalence of contraceptive use (ICF, 2022). Provinces with lowest contraceptive use, East Nusa Tenggara, Maluku, Papua, West Papua are among regions with the highest TFR in Indonesia. However, the association between contraceptive use and TFR is not clearly seen in provinces with high contraceptive use. For example, Central Kalimantan’s TFR is still above replacement level at 2.5 despite of its high contraceptive prevalence rate.

9 See Jones (2005) for estimates on the rising share of never-married women by age groups using estimates drawn from the Census series 1960 to 2000.
